# Ontological insecurity and dual identity: how social change anxiety shapes local-national identification among Hong Kong residents

**DOI:** 10.3389/fpsyg.2026.1821184

**Published:** 2026-08-04

**Authors:** Wanqing Zheng, Sara Akram

**Affiliations:** 1 School of Marxism, Sun Yat-sen University, Guangzhou, China; 2 Center for Studies of Hong Kong, Macao and Pearl River Delta, Sun Yat-sen University, Guangzhou, China; 3 Central China Normal University, Wuhan, China; 4 Department of Law, The University of Faisalabad, Faisalabad, Pakistan

**Keywords:** existential angst, Hong Kong residents' identity, local-national identification, ontological insecurity, social change, the dual identity

## Abstract

Dual identity people believe that they are a part of a local community and national group at the same time. Past studies have indicated that individuals who have dual identities are likely to decrease intergroup conflict, whereas those with a single identity are likely to increase social divisions. In Hong Kong, there are numerous inhabitants that consider themselves as both Hongkonger and Chinese. The continued social changes have however questioned how these changes have been influenced by these changes on dual identity. In this study, three questions are covered. First, does it have a concern about social change as it pertains to dual identity of the Hong Kong residents? Second, is the experience of personal life predictor of the less desirable impact of such worry on dual identity? Third, do these relationships vary based on the age of a person or whether he or she was born in Hong Kong or another place? Information was sampled using a giant and representative cross-sectional survey of the adults of Hong Kong. Dual identity, existential anxiety, life stability, and sense of incompatibility were measured using standard measures. Direct relationships and interaction effects by age and place of birth were investigated using regression models. The results indicate that anxiety about social change had a negative correlation with dual identity particularly among the residents aged 35 and above. This relation was not found to be significant between younger residents. The sense of stability in everyday life had a positive correlation with dual identity and minimized the adverse impact of social change worry on various groups. Social change concern was a significant predictor of weaker dual identity in the aged residents. The personal stability and sense of incompatibility among the younger and locally-born residents were more correlated with dual identity than with social change.

## 1 Introduction

In the past several decades, research has been done on how the identities of residents of Hong Kong affect their development outlook of their country, as well as their relations with the mainland. Although this has been a popular subject among academics and politicians, many studies fail to fully address the basic theories of identity formation. The greatest of these misconceptions is framing the Hong Kong identity and the Chinese identity as mutually exclusive. Such gross oversimplification misses the many lived realities of a person's sense of belonging and the intricate social relations present in the case of the Hong Kong society.

It is a misconception held by many that a person of Hong Kong Chinese descent cannot possess a dual identity as a Hongkonger and a Chinese person (Fong, 2017; Lam, 2004; Qiang, 2014). From the standpoint of the negative view, this perspective fails to recognize that Hong Kong is part of China, both spatially and legally (Mathews et al., 2008). A local identity and a national identity will naturally complement each other when there is no political conflict. While people can easily possess multiple identities, the situation where local and national identities are politically defined is viewed predominantly from the perspective of a political conflict, the scenario where the identities coalesce is ignored (Ma and Fung, 1999).

The surveys carried out in a range of universities in Hong Kong show that a significant percentage of the respondents identified themselves as both Hongkongers and Chinese (Lau and Kuan, 1988; Ma, 1999; Kim and Ng, 2008). These results indicate that dual identity is not something extremely uncommon or even impossible among Hongkongers, but they do not reflect the opinion of all Hongkongers. It is interesting that in political debates, people are more likely to pick both identities and not just one, instead of forcing them to choose between the two (Veg, 2017). This contradicts the argument that political debates divide people's loyalties.

An additional problematic view regarding identity formation is what is referred to as the “single identity theory,” which asserts that individuals can only hold one dominant identity at a time (Tajfel and Turner, 1979). This theory does not adequately reflect the reality of Hong Kong, where the social and political context makes multiple layered identities both natural and common (Ku, 2012; Fung, 2001; Wu, 2011). Due to its uniqueness as a Special Administrative Region (SAR) of China, and the “one country, two systems” policy, residents of Hong Kong are provided with structural justification to possess and express multiple social identities (Chan, 2014). Socially ascribing individuals to a limited number of identity categories is counterproductive, as it increases social fragmentation and the potential for social discord, while simultaneously reducing the level of social interplay and integration that is crucial for the welfare of society as a whole (Cheng, 2019).

Favoring dual identity is not merely a political choice (Guo, 2013). Social psychology studies have always indicated that individuals who have more than one social identity possess a better ability to deal with intergroup tensions, have a greater understanding of others (Jiang, 2016), and contribute more positively to social harmony (Gaertner and Dovidio, 2000; Roccas and Brewer, 2002). In such a society as Hong Kong where the dual identity has become a sensitive political issue, knowing what can enhance or undermine dual identity is of real value to both the social wellbeing and the policy of the people (Amiot et al., 2007; van Dommelen et al., 2015; Verkuyten and Pouliasi, 2006). This study investigates how social changes impact the dual identities of residents in Hong Kong. It examines three questions. First, does social change apprehension diminish the dual identity holding capacity? Do residents who feel uncomfortable social changes consider themselves less Hong Kong and Chinese? Second, does personal stability impact dual identity positively and diminish change related apprehension? In uncertain times, does personal stability enable individuals to preserve both identities? Finally, are there any differences based on age and place of birth in the aforementioned relationships? Do younger and older people feel and experience these relationships differently? Do people born in Hong Kong experience these relationships differently than those born outside? To promote social cohesion in Hong Kong, it is imperative to understand these elements in residents' lived experiences, avoiding political simplifications, and addressing the changing society and shifting complex identities of the residents.

### 1.1 Identity theory and dual identities

Identity signifies the understanding of the self, as well as the understanding of the self as defined by others. There are two sides to identity. The first is objective, which consists of structural components like legal or social identifications as part of a group. The other, the subjective side of identity, includes feelings of emotional attachment to a group, in addition to the social and psychological sense of belonging. This duality reinforces the idea that identity is a personal and emotional experience that the individual goes through rather than a socio-political phenomenon placed on a person by a society (Jenkins, 2014; Castells, 2010).

Identity consists of three distinct components. The first is sameness, which includes traits that are stabilized and invariable throughout time. The second is belonging, which consists of the emotional attachment an individual feels toward a group. The last is recognition and approval, which stresses the significance of the individual being socially recognized (Brubaker and Cooper, 2000). These components of identity assist in addressing the fundamental concerns of an individual or a collective, which are, \“Who am I?\” and \“Who are we?\” The myriad of possible responses have invited different theorists to offer distinct explanations (Hall, 1996).

Identity is seen from a primordial viewpoint as something passed down through one's history, culture, or ethnic background, and therefore unchangeable. Conversely, from a constructivist standpoint, identity is malleable and shaped by prevailing social conditions and historical context. Identity, in social terms, is a perpetually negotiated construct, subject to unending change through social interactions. Identity is also social and means participatory (Anderson, 1983). Strategic theory stands in the middle of the continuum and posits that identities can change, but that change is largely contingent on existing social circumstances and cultural frameworks (Cornell and Hartmann, 2007).

With respect to developing a theory of identity, both constructivist and strategic theories emphasize that an individual can assume several identities at the same time. Psychological research supports this as people often construct an identity that encompasses varying dimensions rather than simply fostering an identity that is singular and exclusive (Roccas and Brewer, 2002). Different identities can coexist and even function in an integrated manner and this is often the case where the identities are not in conflict (Hogg and Abrams, 1988). Multiple identities can also differ in strength and relevance from one situation to the next.

In social and political thinking, the idea of single identity is dominant and is often associated with social conflicts (Smith, 1986). Tight and rigid group boundaries create more inter-group competition and worsen division in the social group along the lines of “us” vs. “them” (Tajfel and Turner, 1979). Amartya Sen notes that a strong exclusive identity increases social distance, and can even trigger animosity and violence (Sen, 2006). Narrowing of identity boundaries makes inter-group communication more difficult (Geertz, 1973), and makes conflict resolution even more difficult. This results in a state of “self-enclosed hostility” where polarized hostility is directed at one another by the groups (Putnam, 2007). On the other hand, people who carry multiple and more inclusive social identities can diminish social antagonism and hostility and foster inter-group communication (Gaertner and Dovidio, 2000; Hobsbawm and Ranger, 1983).

In the case of Hong Kong, the narratives of exclusive identities that compel people to identify only as “Hongkonger” or “Chinese” are likely to exacerbate the conflict between Hong Kong and mainland China (Fong, 2017; Hogg and Abrams, 2010). Inclusive identity as “Hongkonger” and “Chinese” is essential to establishing a basis of understanding and is viewed positively (Veg, 2017). Studies that exclusively focus on either national or local identity are not as useful (Mathews et al., 2008). Additionally, most of the research has focused on political or historical explanations, while the psychological impact of social change, including anxiety, has largely been overlooked (Lam, 2004).

The study analyses Giddens\' theory of modernity and self-identity. Giddens describes modernity by the process of “disembodying” whereby social relations are detached from local settings and subjected to the influences of remote factors, such as globalization (Giddens, 1990, 1991b). Despite the fact that the people of Hong Kong are intricately connected to global processes (Sen, 2009), people retain their local attachments. Disembodying processes cultivate uncertainty, disrupt the routines people rely on, and propel the pursuit of ontological security—the confidence people have in the persistence of their self-identity and the social context that surrounds them. When people lose confidence in their social context (Pruitt and Kim, 2013), they experience existential anxiety as a result of the uncertainty and disruptions (Giddens, 1991a; Kinnvall, 2004).

In the process of rapid social transformation, individuals attempt to establish order by making deliberate and strategic choices with respect to their identities. The residents of Hong Kong have experienced quite considerable pressures such as the rapid changes in the economic sphere, the development of the political relations between Hong Kong and mainland China, and the increase in inequality (Li, 2005; Liu, 2000). In this case, the researchers have reported a rise in the levels of anxiety among the residents with respect to the future of the city (Ku, 2012). For some people, the anxiety led shifted from the inclusive dual identity toward a more exclusive local identity (Fong, 2017; Cheng, 2019; Giddens, 1998, 2011). Relatively little has been done to understand the relationship between anxiety, the desire for stability in life, and the dual identity. This is a worthy research question that will direct focus to the identity dynamics in current Hong Kong. Existential anxiety is a profound sense of existential anxiety that occurs when individuals feel that the social conditions that they are relying on to provide them with a stable sense of self are under threat (Giddens, 1991a). It is not the same as everyday stresses since it brings more serious questions concerning the identity of a person and his/her place (Li, 2016; Li and Zheng, 2016; Pang and Jiang, 2015). The emergence of rapid political and social changes has placed Hong Kong in a situation where its residents may be uncertain about the future of their way of life, their rights and their cultural identities. Such uncertainty can not only impact on the personal wellbeing of the individuals, but also how individuals define their social identities. Although it is a significant topic, there is very little research that has directly examined the relationship that exists between existential anxiety and dual identity in Hong Kong. The proposed study fills this gap by considering the concept of existential anxiety as a central variable and analyzing the mechanisms of action in conjunction with personal life stability and demographic background to develop dual identity.

In this paper, the author focuses on the relationship between social changes and dual identities of Hong Kong residents. It answers three key questions. First, does the social change worry about weaker dual identity with Hong Kong residents? That is, are concerned residents who are worried about social changes less connected to their Hong Kong and Chinese identity? Second, does the sense of personal life stability to sustain dual identity and alleviate the adverse impact of social change worry? Third, are these relationships varied according to the age of a person or his/her place of origin, i.e. whether he was born in Hong Kong or elsewhere?

## 2 Methodology

### 2.1 Research design

This paper will use a quantitative and cross-sectional design research that intends to analyze the relationship between existential anxiety and dual identity among the residents of Hong Kong. The third wave of Hong Kong Panel Study of Social Dynamics (HKPSSD) was used as the source of data. This research takes two parallel samples to determine whether the results can be replicated in various means of measuring dual identity. The sample was divided into two groups: the first group was the control group that measured dual identity as a continuous variable with a numeric scale. The second sample was the experimental group which measured dual identity as a categorical variable with three identity types. This two-sample design was intentionally done to determine whether the findings are valid irrespective of the measure of dual identity. The fact that two different methods of measurement have been used enhances the reliability of the findings since similar results were obtained using the two different methods of measurement. The researches address the question of whether the correlation between existential anxiety and dual identity can be considered the same when dual identity is measured differently. Here it should be noted that the terms are used here in a measurement sense and not in the sense of an experiment with random assignment to treatments. The two groups were slightly different versions of the dual identity measure, and by comparing the results between the two groups, we can test the hypothesis that the results depend on the specific version used to measure the dual identity. The fact that the results of the two groups are consistent raises the confidence in the overall conclusions of the study.

### 2.2 Data source and sample

The paper will be based on the findings of the Hong Kong Panel Study of Social Dynamics (HKPSSD) which was a large-scale household follow-up survey conducted by the Hong Kong University of Science and Technology professor, Wu Xiaogang. The survey uses computer assisted interviews and data were collected in 2011, 2013, and 2015. The required questions on the desired and anticipated future political systems that are at the heart of the existence anxiety are only found in the third wave. Hence, this paper will only use the third-wave data. There are 5,160 adult respondents in the total sample. In the latter, there are 2,488 respondents in the control group and 2,575 respondents in the experimental group. Stratified random sampling was used to make the sample representative of the Hong Kong adult population. In this method, the populace was initially segregated into subgroups as far as key features like age, gender, and location of residence were concerned. Each subgroup was then randomly chosen the number of households in each subgroup relative to the household share of the total population. This approach also allows the representation of the various segments of the Hong Kong population in the sample to be fair, thus enhancing the accuracy and generalizability of the results. This sample provided all the variables that were used in this study thereby giving consistency in measures.

The study relied on secondary data taken under the permission of an institutional ethics committee in Hong Kong in the Hong Kong Panel Study of Social Dynamics (HKPSSD). Since this research entails a survey data analysis of anonymized data, no further ethical approval was necessary. Informed consent was given by all participants in the initial survey before they participated. Their personal data was completely anonymized, and no identifying data were employed in this research. Access and use of the data were in compliance with the terms of the original data providers. Descriptive statistics of all variables are presented in Table 1.

**Table 1:** Descriptive statistics of variables.

Variable	Dual identity (continuous measure)			Dual identity (categorical measure)		
	*N*	Mean	SD	*N*	Mean/count	SD
Degree of dual identity	2,488	28.36	13.57	2,579	1,745/399/435	
Expected socio-political environment	1,726	5.89	2.33	1,751	5.92	2.41
Expected most likely socio-political environment in 2025	1,726	4.83	1.85	1,751	4.86	1.86
Difference between expected and desired environment in 2025	1,726	−1.06	2.23	1,751	−1.06	2.23
Uncertainty about socio-political environment in 2025	1,726	2.97	0.78	1,751	2.97	0.83
Prediction of personal situation in 5 years	2,488	2.97	0.64	2,579	2.99	0.65
Participation in social movements in past 5 years	2,488	0.08	0.26	2,579	0.08	0.28
Internet as main news source	2,488	0.27	0.44	2,579	0.27	0.44
Income	2,317	10,378.98	15,621.86	2,387	10,011.20	12,155.01
Log of income	2,317	8.05	2.09	2,387	8.02	2.11
Years of education	2,473	9.69	4.20	2,563	9.56	4.45
Age	2,488	49.26	19.15	2,579	50.04	18.89
Gender (male/female)	2,488	1,172/1,316		2,579	1,225/1,354	
Place of birth (HK/mainland/others)	2,488	1,462/945/81		2,575	1,520/972/83	

For categorical variables (Gender and Place of birth), values are presented as counts. For degree of dual identity in the categorical measure, values represent counts of dual identity (1,745), Hong Kong identity only (399), and Chinese identity only (435). As demonstrated in this table, the control and experimental samples are very close in all the important variables, such as age, gender, income, education, and place of birth. This comparability establishes that the two samples are well matched and that the differences in results between the two groups is not a result of differences in the samples.

## 3 Measures and variable operationalization

### 3.1 Dual identity

Previous researchers usually determined dual identity by asking the question: whether one had two identities simultaneously. This informs us of the kind of identity and not the intensity of person identity. This study employed a product method in order to capture type and strength. The third wave of Hong Kong Panel Study of Social Dynamics (HKPSSD) was used as a source of data. The respondents in the control group were asked to rate the degree to which they identified themselves as being Chinese or as being Hongkonger on a 0–7 scale. We took the product of the two scores, and that made 49 potential values which correspond to varying degrees of dual identity strength. However, given that different pairs of scores can produce the same product (e.g., 2 x 2 = 1 x 4 = 4), these were infrequent—only 1.45 percent of the sample. The experimental group was measured by a dissimilar scale and responses were classified into three groups; mostly, Hongkonger, mixed, or mostly, Chinese. To verify that our findings were consistent, we conducted analysis of the continuous and the grouped measures. This is a more comprehensive view of Hong Kong dual identity.

### 3.2 Existential anxiety

Existential anxiety as defined by Giddens comes in whereby, the speed of social change causes people to be confused about themselves and whether their world is changing or not (Hui and Xu, 2017; Zhao et al., 2005). It is not like daily concern. It is about the more profound questions such as the ones asking whether I remain myself, and what I will be. Ethnographic descriptions of Hong Kong residents and past qualitative research have documented anxieties about losing the unique character of the city and its voice amidst increasing ties with mainland China (Ku, 2012; Fong, 2017). Others were not sure of the pace at which change was occurring. We used data of HKPSSD to measure this anxiety. We examined the distance between the desired and anticipated political system of people in 2025 and 2050. The wider distance is a red flag of more anxiety regarding the loss of uniqueness and control. We also determined the confidence people had on future political arrangements. Reduced confidence is an indicator of increased uncertainty. Combined, these indicators reflect two important aspects of existential anxiety: the fear of losing the unique features of Hong Kong and the fear of having an unpredictable future.

### 3.3 Other independent and control variables

Existential anxiety encompasses the topic of general social change but can have an impact on identity via personal experience. So we captured the feelings that people have toward their daily lives both good feelings such as stability and bad feelings such as powerlessness. To categorize these feelings into measurable items we applied the factor analysis on HKPSSD data. We as well added the extent to which people were optimistic about their own future as worry about the society may also be transferred into personal outlook. The issue of media as well: individuals who primarily obtain news through the Internet could have a different view of social change. The presence of previous involvement in protests was also covered, as the extensive identification with one group may result in collective action. Lastly, we included common controls, such as age, sex, birthplace, education, and income. These variables are important to us since including them would make us realize whether the anxiety of existentialism is really having its way on dual identity or whether other variables were working.

### 3.4 Data analysis strategy

Stata 13.1 was used in carrying out analyses. In the case of control group sample, where a measurement of dual identity is in the form of a continuous variable with a range of 1 to 49, ordinary least squares (OLS) regression was used. This model approximates the impact of existential anxiety and other independent variables toward the extent of the dual identity. In the case of the experimental group sample, where dual identity is a three-category variable (Hongkonger, mixed, Chinese), multinomial logistic regression (Mlogit) was adopted. According to this model, the likelihood of belonging to each of the identity categories is estimated with respect to a baseline based on the influence of existential anxiety and other variables. The study attempts to determine whether the association between existential anxiety and dual identity is strong by using two regression models and a sample of two cases of two different measurement and modeling methods. The control variables such as the demographic characteristics, individual perceptions of life, media use, and participation in protests were entered in the regression models in sequential blocks. This block entry method will enable us to see how the effect of the existential anxiety on the dual identity changes as each set of control variables is introduced. This will assist in finding out which variables explain some of the relationship between existential anxiety and dual identity. It should be mentioned that this method analyses the statistical control and buffering of these variables in some regression framework, as opposed to formal mediation or moderation analysis.

## 4 Results and findings

### 4.1 Research question 1: is worry about social change associated with dual identity?

Regression analyses showed that existential anxiety has a significant negative effect on dual identity of Hong Kong citizens. Table 2 shows the results of OLS regression of the control group sample. There are four different models, 1 through 4, where the independent variables are added one by one. Once existential anxiety measures have been incorporated, Model 2 accounts for 1.7% more variance than Model 1; Model 4 accounts for 2.5% more variance than Model 1, both of which are significant additional exploratory power. Existential anxiety was assessed on two dimensions. To begin with, the variation between the preferred political system of the respondents and the one they anticipated in 2025 and 2050. With every one-unit change in this gap, the dual identity scores reduced by 0.548 (Model 2) and were significant within all the models. Second, the effects of uncertainty about future political arrangements were even greater: every one unit increase in uncertainty decreased dual identity by about 2.5 points with all models. These are the findings that support both Hypotheses 1 and 2. Sense of life stability was also positively related with dual identity among life perception variables (b = 1.163, *p* < 0.01). Amazingly, tension also had a positive impact (b = 2.669, *p* < 0.01) indicating that not all negative feelings undermine dual identity. Feeling of helplessness and panic did not play a major role. The main source of news Internet was positively related with dual identity (b = 1.678, *p* < 0.05). Hypothesis 7 received strong support since participation in social movements in the last 5 years had a negative impact (b = −5.441, *p* < 0.01). These trends were substantiated by robustness checks by multinomial logistic regression on the sample of the experimental group. Increasing inequality between desired and anticipated forms of political system greatly diminished the chances of having mixed identity, which means that respondents were more likely to see themselves as either purely Hongkonger or purely Chinese. Each of the models adjusted income, education, age, gender, and birthplace (see Table 2 for details).

**Table 2:** Existential anxiety and dual identity (continuous measure).

Variables	(1)		(2)		(3)		(4)	
Existential anxiety								
Discrepancy (expected vs. desired)			−0.548^***^	(0.151)	−0.544^***^	(0.150)	−0.619^***^	(0.152)
Uncertainty about prediction			−2.693^***^	(0.435)	−2.561^***^	(0.435)	−2.510^***^	(0.432)
Individual life perceptions								
Sense of life stability					1.125^**^	(0.441)	1.163^***^	(0.438)
Sense of powerlessness					−0.681	(0.545)	−0.520	(0.543)
Panic					−0.104	(0.721)	−0.004	(0.716)
Tension					2.435^***^	(0.852)	2.669^***^	(0.849)
Prediction (5 years)					−0.028	(0.517)	−0.070	(0.516)
Internet and social movements								
Internet as main news source							1.678^**^	(0.802)
Participated in social movements (5 years)							−5.441^***^	(1.286)
Control variables	Yes		Yes		Yes		Yes	
Constant	24.472^***^	(1.354)	31.620^***^	(2.832)	31.254^***^	(3.067)	31.154^***^	(3.128)
Observations	5,386		1,569		1,569		1,569	
R-squared	0.042		0.059		0.071		0.085	

OLS regression coefficients and standard errors in parentheses. The control variables are income, years of education, age, gender, and place of birth. *N* = 1,569. *R*^2^ = 0.085 for the full model.

^*^p < 0.10. ^**^p < 0.05. ^***^p < 0.01.

Table 2 contains the results of ordinary least squares regression that investigates the impact of existential anxiety on dual identity as a continuous measure (0 49 scale). Models 1, 2, 3, and 4 are intermediately increasing the blocks of variables. Model 1 contains control variables only. Model 2 incorporates the two measures of existential anxiety. Both exhibit a lot of adverse outcomes. Dual identity decreases by 0.548 (*p* < 0.01) with every increase in the disparity between anticipated and preferable political systems. There is a negative correlation between dual identity and each unit change in uncertainty about future political arrangements, with a value of 2.693 (*p* < 0.01). Model 3 includes personal life perceptions. Dual identity has a positive relationship with life stability (b 1.125, *p* < 0.05). A positive effect is also demonstrated by tension (b = 2.435, *p* < 0.01). It does not matter whether there is powerlessness and panic. Model 4 is an addition of internet use and protest participation. The use of internet as the primary news source is positively correlated to dual identity (b = 1.678, *p* < 0.05; Liu, 2013, 2014; Xue et al., 2013; Zheng and Li, 2017). There is a strong negative influence (b = −5.441, *p* < 0.01) on the participation of protests. The entire model describes 8.5 percent of distinct identity variance. The control variables are income, education, age, gender, and birthplace. Parentheses contain the standard errors. Significance levels: ^*^p < 0.10, ^**^p < 0.05, ^***^p < 0.01.

Table 3 describes the results of multinomial logistic regression to investigate the relationship between existential anxiety and identity type. The lowest level is dual identity. The coefficients indicate the probability of having Hongkonger- salient identity or Chinese-salient identity compared to having a mixed identity. In Model 1, there are variables of existential anxiety and life perception. The measure of discrepancy is a great indication that chances of Hongkonger-salient identity are minimal (b = −0.085, *p* < 0.01). It fails to influence Chinese-salient identity significantly. There are no significant impacts of uncertainty of future political arrangement. Life stability has a small negative effect on Hongkonger-salient identity (b = −0.142, *p* < 0.10). Powerlessness has positive effects on Hongkonger-salient identity (b = 0.192, *p* < 0.10) and negative effects on Chinese-salient identity (b = −0.490, *p* < 0.01). The optimism toward the personal future decreases the Hongkonger-salient identity (b = −0.249, *p* < 0.05). Model 2 includes internet use and protest participation. There is an augmentation of likelihood of Hongkonger-salient identity with the use of internet (b = 0.398, *p* < 0.05). Hongkonger-salient identity is strongly predicted by protest participation (b = 0.799, *p* < 0.01). The variables that are used as controls are income, education, age, and gender, as well as place of birth. Parentheses the errors are standard. Significance levels: ^*^ *p* < 0.10, ^**^ *p* < 0.05, ^***^ *p* < 0.01 (see Table 3 for details).

**Table 3:** Existential anxiety and identity types (baseline: dual identity).

Variables	Model 1		Model 2	
	“Hong Kong” salient	“Chinese” salient	“Hong Kong” salient	“Chinese” salient
Existential anxiety				
Discrepancy (expected vs. desired)	−0.085^***^	−0.002	−0.063^**^	−0.003
	(0.030)	(0.034)	(0.030)	(0.035)
Uncertainty about prediction	0.020	−0.010	−0.006	−0.009
	(0.084)	(0.091)	(0.085)	(0.091)
Individual life perceptions				
Sense of life stability	−0.142^*^	0.038	−0.152^*^	0.038
	(0.085)	(0.093)	(0.086)	(0.093)
Sense of powerlessness	0.192^*^	−0.490^***^	0.177^*^	−0.488^***^
	(0.106)	(0.185)	(0.107)	(0.186)
Panic	0.171	−0.126	0.173	−0.122
	(0.145)	(0.203)	(0.146)	(0.204)
Tension	−0.109	0.240	−0.179	0.237
	(0.175)	(0.205)	(0.180)	(0.205)
Prediction (5 years)	−0.249^**^	0.133	−0.202^*^	0.126
	(0.103)	(0.113)	(0.105)	(0.114)
Other moderating variables				
Internet as main news source			0.398^**^	0.081
			(0.160)	(0.184)
Participated in social movements (5 years)		0.799^***^	0.799^***^	−0.341
		(0.189)	(0.189)	(0.314)
Control variables	Yes	Yes	Yes	Yes
Constant	0.883	−2.556^***^	0.548	−2.549^***^
	(0.618)	(0.682)	(0.633)	(0.698)
Observations	1,574		1,574	
R-squared	0.042		0.059	

Multinomial logistic regression coefficients. Standard errors in parentheses. Control variables include income, years of education, age, gender, and place of birth. Baseline category is dual Identity.

^*^p < 0.10, ^**^p < 0.05, ^***^p < 0.01.

Existential anxiety was negatively correlated with dual identity, among the majority of the subgroups. This association, however, was not statistically significant in younger residents, whose personal life stability was a more important factor. Both of the dimensions, discrepancy, and uncertainty are negative in great ways on the continuous measure. This trend is strong in other measurement methods. On the categorical analysis, the lack of conformity will diminish the possibility of having a mixed identity and shift individuals toward Hongkonger-salient identification.

### 4.2 Research question 2: does personal life stability buffer the negative effect of social change worry

There are mixed effects in perceptions of life. The continuous measure supports Hypothesis 3 as Life stability enhances the dual identity in the continuous measure. Nonetheless, Hypothesis 4 is not confirmed. There is no importance of powerlessness and panic, although tension is an unexpected predictor of stronger dual identity. It is noted that factor-analyzed items on the HKPSSD survey were used to measure panic and tension in the respondents in terms of how and on which emotional states they reported in their day to day lives. The complete survey tool can be obtained out of the original data source at the Hong Kong University of Science and Technology. This can be a manifestation of cognitive load: learners who find it difficult to balance out two identities can develop a higher level of tension. There is no support in hypothesis 5; the personal future outlook does not affect the continuous model.

The Internet usage correlates with dual identity positively in the continuous measure and predicts the Hongkonger-salient identification in the categorical process. Weaker dual identity and stronger Hongkonger-salient identity is well predicted by protest participation, which is Hypothesis 7.

### 4.3 Research question 3: are these relationships age- and place-of-birth-specific?

Subgroup analysis shows significant variation. There is no significant correlation between existential anxiety and dual identity among youth. To them, stability in life is more important. Females, Hongkong born respondents, and high income also indicate more life stability effects. Powerlessness decreases dual identity among the Hong Kong-born respondents only. The effect of tension is concentrated on men, youth, low-income and Hong Kong-born populations. Youth, older adults and Hong Kong born and mainland born respondents have the greatest effects on the participation of protests. Those trends indicate that the best way to improve dual identity should be designed to address certain subgroups.

The results indicate the existence of a negative relationship between existential anxiety and dual identity among most social groups in Hong Kong, with the exception that the relationship was not statistically significant among younger residents (see Table 4 for details).

**Table 4:** Effects of existential anxiety on dual identity by subgroups.

Variables	Full sample	Male	Female	Age ≤ 34	Age ≥35	Income < 15k	Income ≥15k	Born in HK	Born in mainland	News: internet	News: others
Existential anxiety											
Discrepancy (expected vs. desired)	−0.619^***^	−0.677^***^	−0.592^***^	−0.232	−0.870^***^	−0.448^**^	−0.873^***^	−0.484^***^	−0.833^***^	−0.650^***^	−0.653^***^
	(0.152)	(0.230)	(0.203)	(0.246)	(0.194)	(0.190)	(0.257)	(0.177)	(0.314)	(0.237)	(0.202)
Uncertainty about prediction	−2.510^***^	−2.476^***^	−2.557^***^	−0.860	−3.573^***^	−2.811^***^	−1.763^**^	−2.458^***^	−3.236^***^	−1.125	−3.178^***^
	(0.432)	(0.629)	(0.599)	(0.698)	(0.553)	(0.567)	(0.686)	(0.509)	(0.848)	(0.723)	(0.551)
Individual life perceptions											
Sense of life stability	1.163^***^	0.671	1.519^**^	1.496^**^	0.971^*^	1.019^*^	1.634^**^	1.838^***^	0.014	2.362^***^	0.515
	(0.438)	(0.650)	(0.596)	(0.718)	(0.556)	(0.524)	(0.817)	(0.524)	(0.823)	(0.721)	(0.556)
Sense of powerlessness	−0.520	−0.524	−0.496	−0.341	−0.609	−0.151	−2.465^*^	−2.506^***^	0.322	−2.735^*^	−0.295
	(0.543)	(1.031)	(0.643)	(1.108)	(0.632)	(0.617)	(1.272)	(0.831)	(0.816)	(1.466)	(0.601)
Panic	−0.004	1.122	−0.790	−0.491	0.249	0.240	−1.165	0.080	0.234	0.008	−0.263
	(0.716)	(1.121)	(0.936)	(1.255)	(0.897)	(0.896)	(1.258)	(0.855)	(1.416)	(1.332)	(0.872)
Tension	2.669^***^	3.912^***^	2.048^*^	5.580^***^	1.522	2.456^**^	3.584^**^	3.605^***^	1.669	3.382^**^	2.843^***^
	(0.849)	(1.357)	(1.096)	(1.563)	(1.020)	(1.015)	(1.631)	(1.043)	(1.570)	(1.481)	(1.074)
Prediction (5 years)	−0.070	0.338	−0.257	0.682	−0.575	−0.225	0.587	0.949	−1.827^*^	0.753	−0.540
	(0.516)	(0.792)	(0.686)	(0.864)	(0.653)	(0.653)	(0.872)	(0.633)	(0.937)	(0.832)	(0.661)
Internet and social movements											
Internet as main news source	1.678^**^	0.181	2.718^**^	1.840	1.758	1.500	1.270	2.000^**^	0.798	0	0
	(0.802)	(1.164)	(1.113)	(1.157)	(1.117)	(1.078)	(1.226)	(0.898)	(1.796)	(.)	(.)
Participated in social movements	−5.441^***^	−3.448^**^	−7.464^***^	−6.090^***^	−3.140	−5.153^***^	−5.125^***^	−5.824^***^	−4.815	−4.740^***^	−5.768^***^
	(1.162)	(1.584)	(1.724)	(1.425)	(2.118)	(1.532)	(1.824)	(1.256)	(3.162)	(1.468)	(2.020)
Control variables											
Income	0.000	0.000	0.000	0.000^*^	0.000	0.000	0.000	0.000	0.000	0.000	0.000
	(0.000)	(0.000)	(0.000)	(0.000)	(0.000)	(0.000)	(0.000)	(0.000)	(0.000)	(0.000)	(0.000)
Log of income	−0.001	0.931^**^	−0.575^*^	−0.205	0.058	−0.725	4.150	−0.316	0.477	0.078	−0.118
	(0.243)	(0.392)	(0.328)	(0.460)	(0.312)	(0.498)	(4.705)	(0.290)	(0.528)	(0.428)	(0.299)
Years of education	−0.097	−0.173	−0.045	−0.285	−0.004	−0.144	−0.098	−0.144	−0.042	−0.057	−0.125
	(0.126)	(0.188)	(0.171)	(0.237)	(0.154)	(0.164)	(0.215)	(0.155)	(0.231)	(0.225)	(0.154)
Age	0.111^***^	0.047	0.136^***^	0.023	0.169^***^	0.116^***^	0.016	0.118^***^	0.110^*^	0.070	0.109^***^
	(0.031)	(0.046)	(0.042)	(0.132)	(0.063)	(0.037)	(0.062)	(0.036)	(0.060)	(0.055)	(0.038)
Female	0.366	0	0	−0.035	0.777	0.627	0.272	0.325	1.093	1.456	−0.353
	(0.695)	(.)	(.)	(1.082)	(0.932)	(0.913)	(1.174)	(0.805)	(1.469)	(1.104)	(0.910)
Born in mainland	−0.112	0.052	−0.007	0.206	−0.028	−0.734	1.639	0	0	0.378	−0.386
	(0.776)	(1.180)	(1.046)	(1.323)	(0.965)	(0.930)	(1.455)	(.)	(.)	(1.424)	(0.934)
Born in other	−4.302^*^	−4.582	−3.776	−2.502	−3.942	−4.758^*^	−3.537	0	0	−2.149	−4.961^*^
	(2.252)	(3.602)	(2.911)	(3.874)	(2.785)	(2.680)	(4.272)	(.)	(.)	(4.768)	(2.579)
Constant	31.154^***^	26.429^***^	34.209^***^	29.757^***^	31.232^***^	36.665^***^	−7.689	30.509^***^	33.187^***^	24.180^***^	36.627^***^
	(3.128)	(4.650)	(4.289)	(5.021)	(5.260)	(4.256)	(43.677)	(3.790)	(5.860)	(5.017)	(3.982)
Observations	1,569	712	857	596	973	985	584	1,082	451	603	966
R-squared	0.085	0.092	0.100	0.087	0.096	0.097	0.086	0.110	0.083	0.107	0.089

OLS regression coefficients by subgroups. Standard errors in parentheses. All models control for income, log income, years of education, age, gender, and place of birth. (.) indicates the variable is omitted due to collinearity in that specific subgroup.

^*^p < 0.10, ^**^p < 0.05, ^***^p < 0.01.

Table 4 discusses the OLS regression coefficients detailing the effects of existential anxiety, life perceptions, internet usage, and protest participation on dual identity, in addition to how these effects differ by population subgroups. Each coefficient represents the associated change in dual identity score (from 0 to 49) for each 1-unit increase in the independent variable (with standard errors in parentheses). Each model controls for income, education, age, gender, and place of birth.

The negative impact of existential anxiety on dual identity is present for almost every population subgroup, but there is one notable exception. Respondents aged 34 years and younger show no statistical significance in either measure of the discrepancy or the uncertainty. In this demographic, macro-level worry does not diminish dual identity. In contrast, those aged 35 and older display considerable sensitivity, as discrepancy decreases dual identity by 0.87 points (*p* < 0.01) and uncertainty does so by 3.57 points (*p* < 0.01; Lin et al., 2016). This effect is particularly strong for mid and high income earners as well as those not born on the mainland and those who do not depend on the internet for news.

Among women, youth, higher-income earners, respondents born in Hong Kong, and users of internet news, stability in life circumstances is correlated with stronger dual identity. The largest effects are in Internet news users (b = 2.36; *p* < 0.01) and respondents born in Hong Kong (b = 1.84; *p* < 0.01). The feeling of powerlessness diminishes the dual identity phenomenon only in the cases of respondents born in Hong Kong (b = 2.51; *p* < 0.01) and those earning more than 15,000 HKD (b = 2.47; *p* < 0.10) respectively. The phenomenon of dual identity in relation to tension is observed in many social groups, particularly among young (b = 5.58; *p* < 0.01), men (b = 3.91; *p* < 0.01), respondents born in Hong Kong (b = 3.61; *p* < 0.01), and low-income earners (b = 2.46; *p* < 0.05; Li, 2017).

The Internet as a primary source of news is associated with a stronger dual identity in women (b = 2.72; *p* < 0.05) and respondents born in Hong Kong (b = 2.00; *p* < 0.05). Participation in protests is correlated with a weaker dual identity in all social subgroups, with more pronounced effects in women (b = −7.46; *p* < 0.01) and youth (b = −6.09; *p* < 0.01).

These patterns show that there is significant heterogeneity. Existential anxiety operates mostly among older residents. Life stability protects identity erosion for many groups and for many others this is not the case. Powerlessness affects dual identity only when cross-border ties are weaker. Activism reinforces singular identification in almost all segments of the population.

## 5 Discussion

This research has an evident backup to the main argument that existential anxiety caused by perceived social change has been significantly related to weaker dual identity among Hong Kong residents. When individuals are anxious about the future and are unsure of what Hong Kong is becoming then they start to streamline their identity by retaining only one. These results are consistent between various measurement procedures and statistical models and provide the assurance of their validity. This tendency factors into the argument of Giddens (1991a) who states that the absence of ontological security in form of loss of belief in a continuation of social life drives individuals to claim psychological security through simplified systems of identity. The first important finding is that the lack of expected and desired in the Hong Kong political future undermines dual identity directly. Each unit change in this gap results in a 0.55-point decrease in dual identity scores in the case of a look at 10 years, and a 0.5 point decrease in the case of a look at 35 years. This implies that once the residents feel that Hong Kong is not what they desire it to be, they will be less inclined to identify with Hong Kong as well as with China. The same has been reported in other settings where identity is formed in the light of uncertainty in politics. To illustrate the point, the studies on Catalonia revealed that discordance between aspired autonomy and factual political systems enhanced exclusive regional belonging to the detriment of dual Spanish-Catalan belonging (Guibernau, 2000; Serrano, 2013). The second-dimension uncertainty about the future records even greater effects. The unit increase in uncertainty drives down dual identity by about 2.7 points and the ten-year horizon and 2.5 points and 35-year horizon respectively. It is not mere disappointment that people are motivated to identify themselves as individuals but instead uncertainty. These two results affirm the fact that existential anxiety subverts the concept of dual identity as favored by the theory of Kinnvall (2004) that as individuals experience ontological insecurity, they do so by retreating to small identity lines. Dual identity increases by over a point with each unit of increases in perceived life stability.

This optimistic emotion serves to counteract the pernicious influences of existential anxiety, which is in line with the literature that has discovered safe attachment to the environment permits more adaptable and encompassing identity development (Breakwell, 1986). The negative life feelings (powerlessness and panic) did not however portray the anticipated effects though. Interestingly, the relationship of tension and dual identity was positive. This could be a result of cognitive load: with two identities, a person has two sets of expectations to address and those with lower ability to balance their roles may have more tension (Benet-Martínez and Haritatos, 2005). In this respect, tension can be an effect of dual identity and not a cause. There was no significant age effect on personal future outlook, perhaps because the question in the survey surveyed definite good or bad as opposed to uncertainty as an underlying element of existential anxiety (Giddens, 1991a). In residents of ages 35 years and above, manipulate that existential anxiety is a powerful predictor of weaker dual identity. This audience seems to be more receptive to the accelerated social transformations and more concerned about such issues as whether Hong Kong will be what it is and what is going to happen to it. Their nervousness indicates their real fear of not having what is particular to Hong Kong (Fong, 2017). Studies of Quebec in sovereignty discussions revealed the same age distribution trend with the older generation of residents who recalled the periods before changes having more identity anxiety than the younger generation who viewed unsettled conditions as their natural way of being (Quebec Community Groups Network, 2003). Existential anxiety amongst the young people in Hong Kong however does not have any significance in dual identity. In their case, the stability of the personal life is relevant. Dual identity is more likely among young people who live stable lives and not their counterparts who live unstable lives. It implies that younger residents approach social change in a different way, maybe they are more flexible, or maybe macro-level issues are less poignant than the need to address personal issues on a case-by-case basis (Veg, 2017; Ma and Fang, 2019).

There is a different pattern in Hong Kong-born residents. In this case, the perceptions of powerlessness are a direct decrease in dual identity. One unit rise in powerlessness results in a decrease of over two points in their dual identity score. Their personal connections to the mainland are also not strong in comparison to natives of the mainland. They lack the good cross-border relationships that they can rely on when they feel that they cannot do anything about their future or the future of Hong Kong (Mathews et al., 2008). This can enhance their feeling of alienation and lead them to a Hong Kong identity. A similar scenario can be found in Scotland, where native-born Scots who felt politically powerless during the Brexit start to reject dual British-Scottish identity and embrace exclusive Scottish identity more, whereas English-born residents in Scotland are less committed to cross-border connections (Henderson et al., 2016).

Internet use is having mixed impacts. It is a stronger predictor of dual identity in the continuous measure. Hongkonger-salient identification is predicted by it in the categorical measure. This can be an indication of the various content and echo-chambers of the online domain (Sunstein, 2017). The presence of protest, however, is quite obvious and uniform. The participants in the social movements that have been observed in the last 5 years exhibit considerably poorer duality and are far more likely to consider themselves Hongkonger. This trend is the highest among the youth, older people, and respondents born in Hong Kong as well as those born in the mainland. When identity is mobilized into action then it seems to take a single form (Drury and Reicher, 2009). These trends were also evident in the Umbrella Movement in 2014, when the participants of the protests made significant changes in dual to exclusive local identity (Chan and Chan, 2017; Cheng, 2016). The implications of these findings are clear. To a majority of the Hong Kong inhabitants, uncertainty of the future and ability to bridge the expectations and reality would most likely reinforce dual identity. In the case of youth and Hong Kong-born population, it may be more effective to increase the living standards and improve the personal life stability rather than to reassure the macro level only. One size does not fit all. This can be achieved by customized strategies that meet the particular fears and concerns of the various subgroups and this is likely to be the most effective in creating a sense of belonging (Sen, 2006).

The National Social Science Fund of China (NSSFC) supported this study. The authors recognize that the source of funding can bring up a concern on the possibility of an influence on the direction of the research and the findings. Authors confirm that data analysis and interpretation were independent and that the results are based on the statistical outcome of the survey data and not the external agenda. The readers are urged to consider the findings and the conclusion on their own merits.

## 6 Conclusion

The concern regarding socio-political transformations has a negative impact on the dual identity phenomenon among Hongkongers. As such worries and problems intensify, individuals become disillusioned and resort to identity foreclosure. This pattern holds across differing measuring and modeling approaches. Existenatil anxiety diminishes dual idnetity and, more importantly, the impact of anxiety. The impact of stability, however, is not even. Residents of 35 years and older, and who experience existential anxiety, exhibit less dual identity. Younger generations, on the other hand, do not experience existential anxiety, and place the highest value on personal stability, and more so than macro level caused stability. Greater sense of powerlessness is reported by Hong Kongers, which causes reduction of their dual identity. Protest participation nearly universally predicts singular identification across all subgroups. Most residents seeing reaffirming stability around “one country, two systems” and the easing of uncertainty regarding the future of Hong Kong would, most likely, increase their dual identity. For young people, and more so, for those born in Hong Kong, personal life stability and their living conditions is more important than macro-level positive changes. Therefore, targeted approaches to address the specific concerns and needs of different subgroups are highly important.

## 7 Policy recommendations

According to the results of this paper, the following recommendations could assist in supporting dual identity among the Hong Kong residents.: 1. Face up to the fact that Hong Kong residents have multiple identities. Policy strategies that do not strengthen single identity narratives and that limit the politicization of local and national identities may help maintain dual identity among the residents. 2. Pay attention to the role of groups with “patriotism for both mainland China and Hong Kong”. This group has dual identities, as they highly identify themselves as both “Chinese” and “Hongkonger”, which is conducive in conciliating and mediating the conflicts between the two places, maintaining close contact between the two places and cementing their relationship. 3. Bolster and consolidate the confidence of Hong Kong residents in “two systems” and cultivate their “patriotism for both mainland China and Hong Kong”. Existential anxiety is closely related to the pervasive influence of habits feelings in daily life. The design of the “two systems” has ensured Hong Kong residents' daily life stayed unchanged, guaranteed their ontological security and dual identities, and thus they will have more “patriotism for both mainland China and Hong Kong”. Bolstering and consolidating the confidence of Hong Kong residents in “two systems” will help to grasp the public attitude and ensure the smooth implementation of “one country, two systems” in Hong Kong. 4. Abate the uncertainty of social changes in the two places, meanwhile hoisting the dual identities of “patriotism for both mainland China and Hong Kong”. For most Hong Kong residents, reducing the uncertainties of social changes in the two places can elevate their dual identities. For the youth group and the group born in Hong Kong, it is necessary to improve their living standards, enhance their sense of stability and reduce their sense of powerlessness, so that they will have more “patriotism for both mainland China and Hong Kong”. 5. Clarify Hong Kong's role in “Guangdong-Hong Kong-Macao Greater Bay Area (GBA),” and encourage Hong Kong residents to seek work opportunities in the northward, thus building a more inclusive “GBA Identity.” By providing better living and working conditions to younger and locally-born citizens, one may contribute to the enhancement of personal stability among the citizens and, hence, help bolster the dual identity. Given that the Pearl River Delta region borders on Hong Kong with intertwined kinship and similar dialect. The increased cross-border prospects and regional integration can offer more social contexts where a more inclusive regional identity can grow naturally.

## 8 Additional study proposals

First, future research should investigate the systems that current anxiety shifts to identity. Longitudinal studies are needed to determine causal direction. The previously mentioned positive relationship between stress and dual identity warrants further investigation. Additionally, for future studies, there should be a focus on the generation differences in identity formation and the existence, if any, of cohort effect persistence over time. The study of if and how Hong Kong differs from other countries with similar political conditions is needed to understand how Hong Kong impacts other countries in the world. Through qualitative study, the daily practices of individuals with dual identity could be studied. In regard to the findings on the internet for this research, studying the digital world's impact on identity formation and the findings of this research are worth studying further.

## Data Availability

This data was collected by The Hong Kong University of Science and Technology, which holds its copyright. Due to copyright restrictions, we are not authorized to share this data with others. For inquiries regarding relevant matters, please contact https://caeser.hkust.edu.hk/en/pssd.
