# Supplementary material for: Ontological insecurity and dual identity: how social change anxiety shapes local-national identification among Hong Kong residents
Source: Front Psychol. 2026 Aug 4;17:1821184. doi: 10.3389/fpsyg.2026.1821184 (PMC13481216; doi:10.3389/fpsyg.2026.1821184)
Supplement: Supplementary file 1 [file Supplementary_file_1.pdf]

Appendix Table

Appendix Table 1. Existential Anxiety (Mid-term Social Environment Change) and Dual Identity (Continuous Measure)

Appendix Table 1. Existential Anxiety (Mid-term Social Environment Change) and Dual Identity (Continuous Measure)

	(1)		(2)		(3)		(4)	
**Variables**	**Coefficient**	**(SE)**	**Coefficient**	**(SE)**	**Coefficient**	**(SE)**	**Coefficient**	**(SE)**
Dependent Variable: Dual Identity								
Existential Anxiety								
Discrepancy (expected vs. desired)			-0.548***	(0.151)	-0.544***	(0.150)	-0.619***	(0.152)
Uncertainty about prediction			-2.693***	(0.435)	-2.561***	(0.435)	-2.510***	(0.432)
Individual Life Perceptions								
Sense of life stability					1.125**	(0.441)	1.163***	(0.438)
Sense of powerlessness					-0.681	(0.545)	-0.520	(0.543)
Panic					-0.104	(0.721)	-0.004	(0.716)
Tension					2.435***	(0.852)	2.669***	(0.849)
Prediction of personal situation (5 years)					-0.028	(0.517)	-0.070	(0.516)
Internet and Social Movements								
Internet as main news source							1.678**	(0.802)
Participated in social movements (5 years)							-5.441***	(1.162)
Control Variables								
Income	0.000**	(0.000)	0.000	(0.000)	0.000	(0.000)	0.000	(0.000)
Log of income	-0.146	(0.133)	0.030	(0.244)	0.021	(0.245)	-0.001	(0.243)
Years of education	0.038	(0.056)	-0.146	(0.126)	-0.120	(0.126)	-0.097	(0.126)
Age	0.157***	(0.012)	0.108***	(0.028)	0.111***	(0.029)	0.111***	(0.031)
Female (ref: Male)	0.378	(0.378)	0.608	(0.698)	0.492	(0.698)	0.366	(0.695)
Born in Mainland (ref: HK)	-0.414	(0.420)	-0.250	(0.778)	-0.107	(0.776)	-0.112	(0.776)
Born in Other places	-3.479***	(0.990)	-4.529**	(2.261)	-4.594**	(2.264)	-4.302*	(2.252)
Constant	24.472***	(1.354)	31.620***	(2.832)	31.254***	(3.067)	31.154***	(3.128)
Observations	5386		1569		1569		1569	
R-squared	0.042		0.059		0.071		0.085	

*Note: This appendix table presents the full regression results corresponding to Table 2 in the main text, showing all control variable coefficients. Standard errors in parentheses. Reference categories: Male (for gender), Born in Hong Kong (for birthplace). * $p < 0.10$, ** $p < 0.05$, *** $p < 0.01$.*

Appendix Table 2. Existential Anxiety (Long-term Social Environment Change) and Dual Identity (Continuous Measure)

Appendix Table 2. Existential Anxiety (Long-term Social Environment Change) and Dual Identity (Continuous Measure)

	(1)		(2)		(3)		(4)	
**Variables**	**Coefficient**	**(SE)**	**Coefficient**	**(SE)**	**Coefficient**	**(SE)**	**Coefficient**	**(SE)**
Dependent Variable: Dual Identity								
Existential Anxiety (Long-term)								
Discrepancy (expected vs. desired in 2050)		-0.505***	(0.132)	-0.491***	(0.132)	-0.553***	(0.134)	
Uncertainty about prediction (2050)		-2.506***	(0.394)	-2.393***	(0.394)	-2.291***	(0.392)	
Individual Life Perceptions								
Sense of life stability				1.157***	(0.440)	1.194***	(0.437)	
Sense of powerlessness				-0.662	(0.543)	-0.512	(0.541)	
Panic				-0.351	(0.718)	-0.268	(0.714)	
Tension				2.158**	(0.852)	2.402***	(0.849)	
Prediction of personal situation (5 years)				0.058	(0.515)	0.005	(0.515)	
Internet and Social Movements								
Internet as main news source						1.299	(0.802)	
Participated in social movements (5 years)						-5.332***	(1.156)	
Control Variables								
Income	0.000	(0.000)	0.000	(0.000)	0.000	(0.000)	0.000	(0.000)
Log of income	0.003	(0.116)	0.133	(0.243)	0.112	(0.245)	0.093	(0.243)
Years of education	0.063	(0.055)	-0.179	(0.125)	-0.154	(0.125)	-0.125	(0.126)
Age	0.159***	(0.012)	0.105***	(0.028)	0.108***	(0.029)	0.103***	(0.031)
Female (ref: Male)	0.343	(0.378)	0.775	(0.696)	0.658	(0.696)	0.517	(0.694)
Born in Mainland (ref: HK)	-0.466	(0.419)	-0.220	(0.776)	-0.095	(0.775)	-0.117	(0.775)
Born in Other places	-3.536***	(0.990)	-5.001**	(2.249)	-5.043**	(2.252)	-4.842**	(2.242)
Constant	23.417***	(1.274)	30.313***	(2.771)	29.857***	(3.028)	29.876***	(3.093)
Observations	5386		1569		1569		1569	
R-squared	0.042		0.067		0.078		0.091	

*Note: This appendix table presents regression results using long-term (2050) social environment predictions. Standard errors in parentheses. Reference categories: Male (for gender), Born in Hong Kong (for birthplace). * p < 0.10, ** p < 0.05, *** p < 0.01.*

Appendix Table 3. Existential Anxiety and Dual Identity (Categorical Measure)

Appendix Table 3. Existential Anxiety and Dual Identity (Categorical Measure)

	(1)		(2)		(3)		(4)	
**Variables**	**Coef.**	**(SE)**	**Coef.**	**(SE)**	**Coef.**	**(SE)**	**Coef.**	**(SE)**
Multinomial Logistic Regression: Baseline Category = Dual Identity								
Outcome: "Hong Kong" Identity Salient (vs. Dual Identity)								
Existential Anxiety								
Discrepancy (expected vs. desired)			-0.088***	(0.029)	-0.085***	(0.030)	-0.063**	(0.030)
Uncertainty about prediction			0.010	(0.083)	0.020	(0.084)	-0.006	(0.085)
Individual Life Perceptions								
Sense of life stability					-0.142*	(0.085)	-0.152*	(0.086)
Sense of powerlessness					0.192*	(0.106)	0.177*	(0.107)
Panic					0.171	(0.145)	0.173	(0.146)
Tension					-0.109	(0.175)	-0.179	(0.180)
Prediction of personal situation (5 years)					-0.249**	(0.103)	-0.202*	(0.105)
Other Moderating Variables								
Internet as main news source							0.398**	(0.160)
Participated in social movements (5 years)							0.799***	(0.189)
Control Variables								
Income	-0.000	(0.000)	-0.000	(0.000)	-0.000	(0.000)	-0.000	(0.000)
Log of income	-0.019	(0.041)	0.036	(0.052)	0.038	(0.052)	0.026	(0.051)
Years of education	-0.043**	(0.019)	-0.021	(0.025)	-0.027	(0.025)	-0.054**	(0.025)
Age	-0.033***	(0.004)	-0.038***	(0.006)	-0.038***	(0.006)	-0.029***	(0.007)
Female (ref: Male)	0.113	(0.121)	0.158	(0.139)	0.187	(0.141)	0.277*	(0.144)
Born in Mainland (ref: HK)	-0.698***	(0.148)	-0.845***	(0.187)	-0.888***	(0.188)	-0.833***	(0.190)
Born in Other places	0.380	(0.314)	0.528	(0.404)	0.498	(0.407)	0.527	(0.411)
Constant	0.813**	(0.413)	0.172	(0.562)	0.883	(0.618)	0.548	(0.633)
Outcome: "Chinese" Identity Salient (vs. Dual Identity)								
Existential Anxiety								
Discrepancy (expected vs. desired)			-0.004	(0.034)	-0.002	(0.034)	-0.003	(0.035)
Uncertainty about prediction			-0.015	(0.090)	-0.010	(0.091)	-0.009	(0.091)
Individual Life Perceptions								
Sense of life stability					0.038	(0.093)	0.038	(0.093)
Sense of powerlessness					-0.490***	(0.185)	-0.488***	(0.186)
Panic					-0.126	(0.203)	-0.122	(0.204)
Tension					0.240	(0.205)	0.237	(0.205)

Prediction of personal situation (5 years)					0.133	(0.113)	0.126	(0.114)
Other Moderating Variables								
Internet as main news source							0.081	(0.184)
Participated in social movements (5 years)							-0.341	(0.314)
Control Variables								
Income	0.000	(0.000)	0.000	(0.000)	0.000	(0.000)	0.000	(0.000)
Log of income	-0.019	(0.035)	-0.018	(0.050)	-0.028	(0.050)	-0.031	(0.050)
Years of education	0.006	(0.016)	-0.004	(0.024)	-0.004	(0.025)	-0.003	(0.025)
Age	0.012***	(0.004)	0.013*	(0.007)	0.013*	(0.007)	0.013*	(0.007)
Female (ref: Male)	-0.001	(0.113)	-0.061	(0.154)	-0.062	(0.155)	-0.060	(0.156)
Born in Mainland (ref: HK)	0.475***	(0.123)	0.733***	(0.157)	0.738***	(0.157)	0.738***	(0.159)
Born in Other places	0.534*	(0.289)	0.253	(0.511)	0.252	(0.514)	0.273	(0.515)
Constant	-2.157***	(0.409)	-2.103***	(0.621)	-2.556***	(0.682)	-2.549***	(0.698)
Observations	2367		1574		1574		1574	
R-squared	0.042		0.059		0.071		0.085	

Note: Multinomial logistic regression coefficients. Baseline category is Dual Identity. The table presents results for two outcome categories: (1) "Hong Kong" Identity Salient vs. Dual Identity, and (2) "Chinese" Identity Salient vs. Dual Identity. Standard errors in parentheses. Reference categories: Male (for gender), Born in Hong Kong (for birthplace). $* p < 0.10$, $** p < 0.05$, $*** p < 0.01$.
